# Innovative Classification of M‐Shaped Lips for Enhanced Refill Outcomes: A Case Series

**DOI:** 10.1111/jocd.70239

**Published:** 2025-05-26

**Authors:** Dina Moctezuma Villalobos

**Affiliations:** ^1^ Signature Clinics Barcelona Spain; ^2^ Aesthetic Medicine and Integral Rejuvenation Universidad Católica de Valencia Valencia Spain

**Keywords:** classification system, cosmetic outcomes, dermal fillers, lip augmentation, M‐shaped lips

## Abstract

**Background:**

Lips are crucial to facial aesthetics, with fuller lips often deemed more attractive. This preference has led to increased demand for non‐surgical lip augmentation using dermal fillers. However, there is a gap in the literature regarding the identification and management of a particular anatomical variant colloquially known as “M‐shaped lips”, characterized by a central “M” configuration of the upper lip. This anatomical variant presents unique complexities. This study presents the first classification system for M‐shaped lips to guide safer, more predictable treatments.

**Aims:**

To introduce the first classification system for M‐shaped lips, improving diagnostic accuracy, treatment strategies, and minimizing complications in cosmetic lip augmentation.

**Methods:**

A case series of nine patients aged 20–45 with M‐shaped lips seeking cosmetic augmentation were treated with Aliaxin LV, a hyaluronic acid filler, in multiple sessions. The proposed classification system was based on the angle between the vertical and horizontal midlines of the lips, including three grades: mild (≤ 15°), moderate (16°–25°), and severe (> 25°). Injection techniques and filler volumes were tailored according to patient classification using needles and cannulas for precise placement.

**Results:**

The classification system facilitated personalized treatments, resulting in satisfactory aesthetic outcomes without significant complications. All the patients showed improved lip symmetry and volume, with no instances of filler migration or vascular complications.

**Conclusions:**

The findings suggest that this classification system can significantly improve cosmetic results and reduce risks in treatment. Larger studies with more diverse populations and extended follow‐up are needed to validate these findings.

## 1 Introduction

The human face is a central area of aesthetic evaluation, with the lips playing a significant role in the overall perception of beauty. The lips are particularly important, given their central role in expression and communication. In females, fuller lips in relation to facial width and greater vermillion height are perceived as more attractive [1, 2]. The perception of attractiveness resulting from enhancing lip volume and shape has led to a substantial increase in cosmetic interventions, including non‐surgical lip augmentation techniques involving dermal fillers. Nowadays, this is one of the most common aesthetic procedures performed worldwide [3].

**FIGURE 1:**
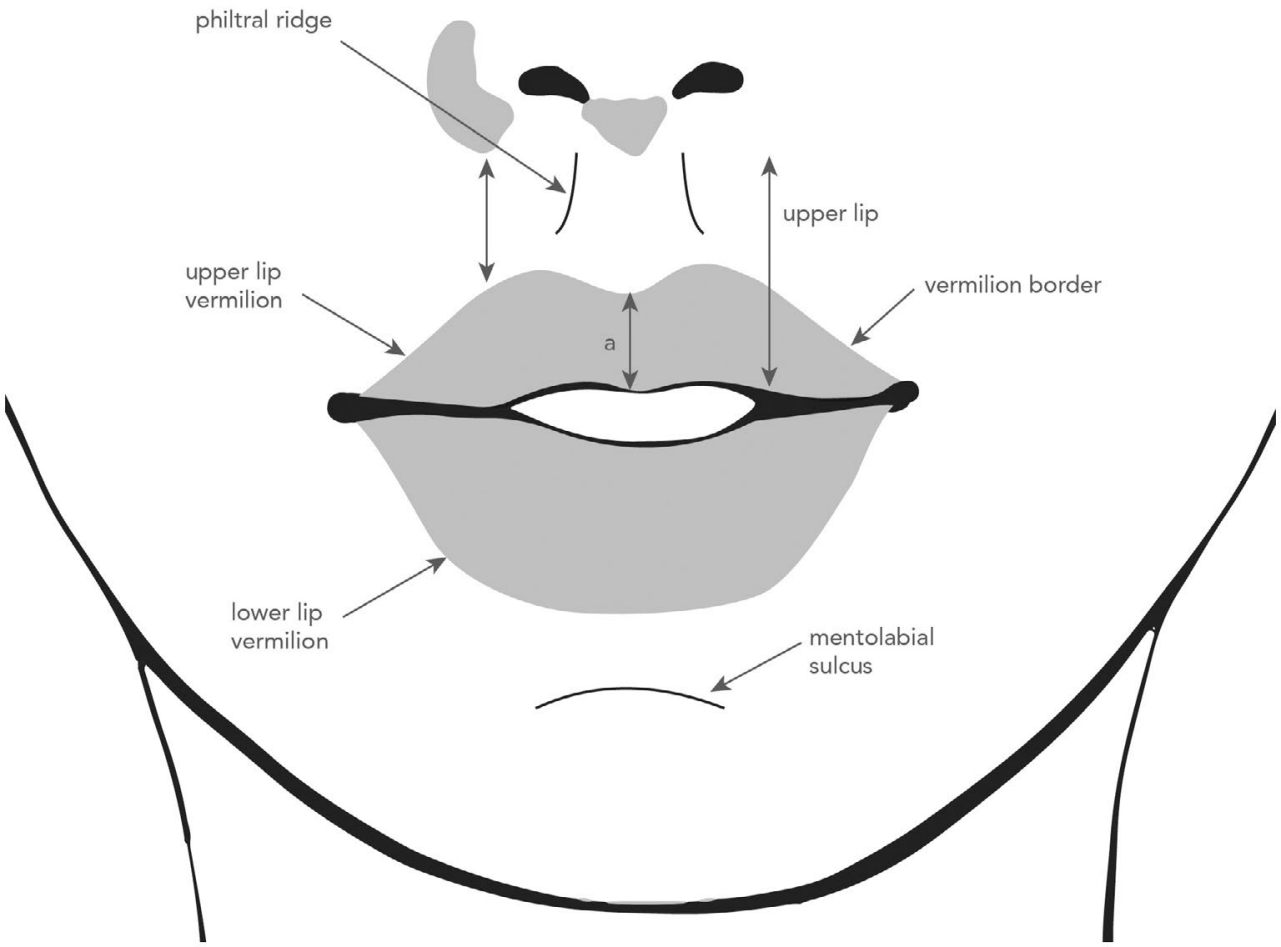
Diagram of lip anatomy.

Lip augmentation requires a minimally invasive procedure that, like any surgical procedure, is not without risks [4, 5, 6]. A basic understanding of the relevant anatomic features of the lips is necessary to achieve the optimal cosmetic and aesthetic outcome (Figure 1). Their intricate vascular anatomy, with superior/inferior labial arteries close to injection sites [7, 8], poses a risk for severe complications, such as vascular occlusion, which, though rare, can lead to catastrophic outcomes like vision loss [4, 5].

**FIGURE 2:**
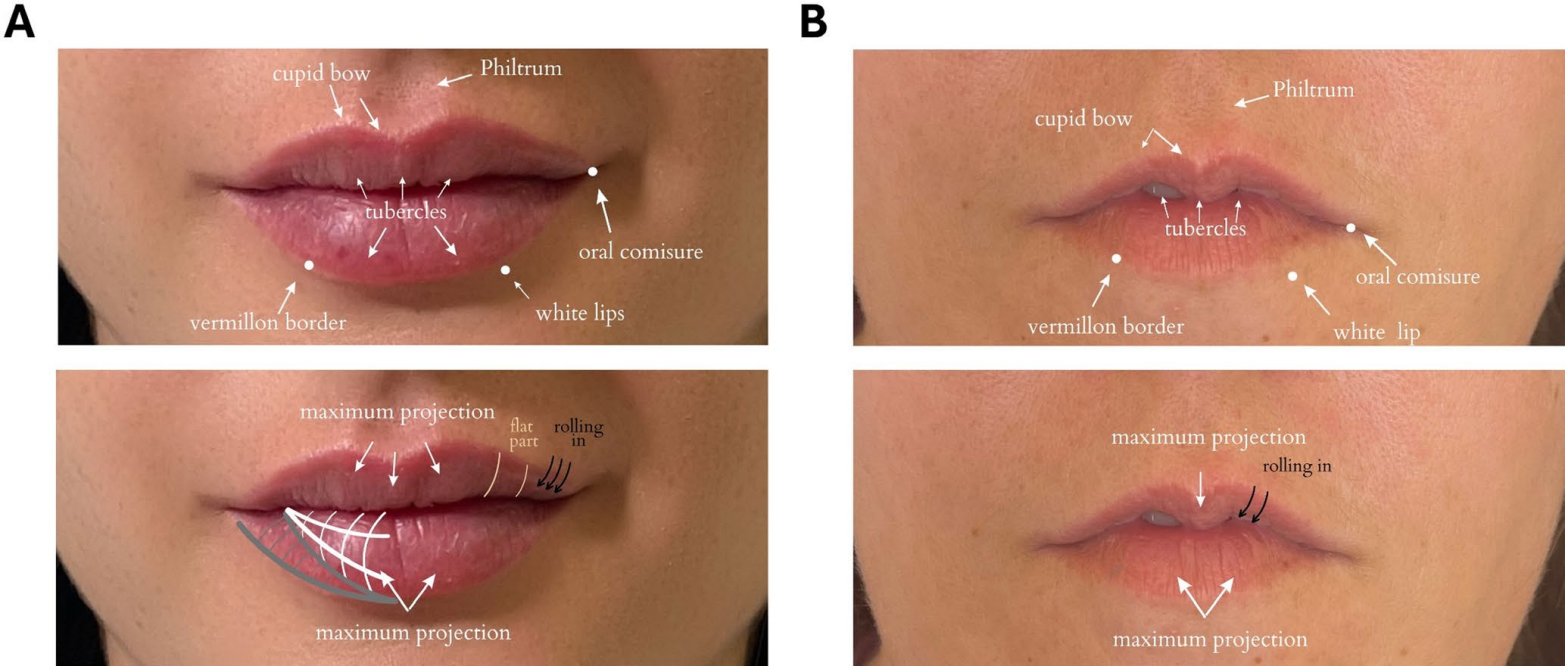
Anatomical comparison between normal‐shaped lips (A) and M‐shaped lips (B).

One of the specific challenges in cosmetic lip enhancement is the treatment of M‐shaped lips [1, 9, 10]. This lip shape is distinguished by an upper lip that resembles the letter ‘M,’ making cosmetic enhancement particularly difficult due to the downward curling of the upper lip muscles and complex anatomical factors [11]. This shape occurs when there is more tissue in the center of the upper lip, with thinner outer portions or inward flipping during smiling (Figure 2). Additionally, the curl or flip of the upper lip tissue can be over‐accentuated, complicating straightforward augmentation. The severity of the M shape can vary significantly, and multiple treatment sessions are usually needed to correct it. As aesthetic expectations in lip augmentation interventions continue to grow, a structured approach to M‐shaped lips is required to minimize potential risks and ensure an optimal outcome [12].

Since M‐shape is not a recognized clinical term, current classification systems for lip shapes do not provide specific guidance for managing this complex lip variant. Classifying M‐shaped lips is crucial for several reasons. First, it allows clinicians to tailor treatment strategies to the patient's specific anatomical needs. This personalized approach ensures that filler materials are placed accurately, minimizing the risk of complications such as filler migration or vascular occlusion. Additionally, by categorizing the severity of the M‐shape, clinicians can select the appropriate techniques and determine the number of sessions required, ultimately leading to more predictable and harmonious results.

To address this need, we developed a novel classification system for M‐shaped lips based on their severity. In this study, we used this classification in a case series of nine patients treated with Aliaxin LV, a hyaluronic acid (HA) filler, with clinical evaluation of the aesthetic outcomes and patient satisfaction following the treatment based on the classification. The new system aims to improve diagnosis and facilitate cosmetic treatment. It is designed to enhance the precision of cosmetic interventions and minimize the risk of complications. By providing guidelines for the appropriate use of techniques and materials, the new classification aims to enhance patient satisfaction and safety and assist cosmetic clinicians in the effective management of M‐shaped lips.

## 2 Methods

### 2.1 Participants

The study group comprised nine patients with M‐shaped lips requesting cosmetic lip augmentation. Patients were included if they were older than 18 years, of both genders, with no previous severe adverse reactions to dermal fillers. Exclusion criteria included active lip infections, known allergies to filler components, previous permanent fillers, and systemic conditions that contraindicate minimally invasive cosmetic procedures.

### 2.2 Classification System for M‐Shaped Lips

**FIGURE 3:**
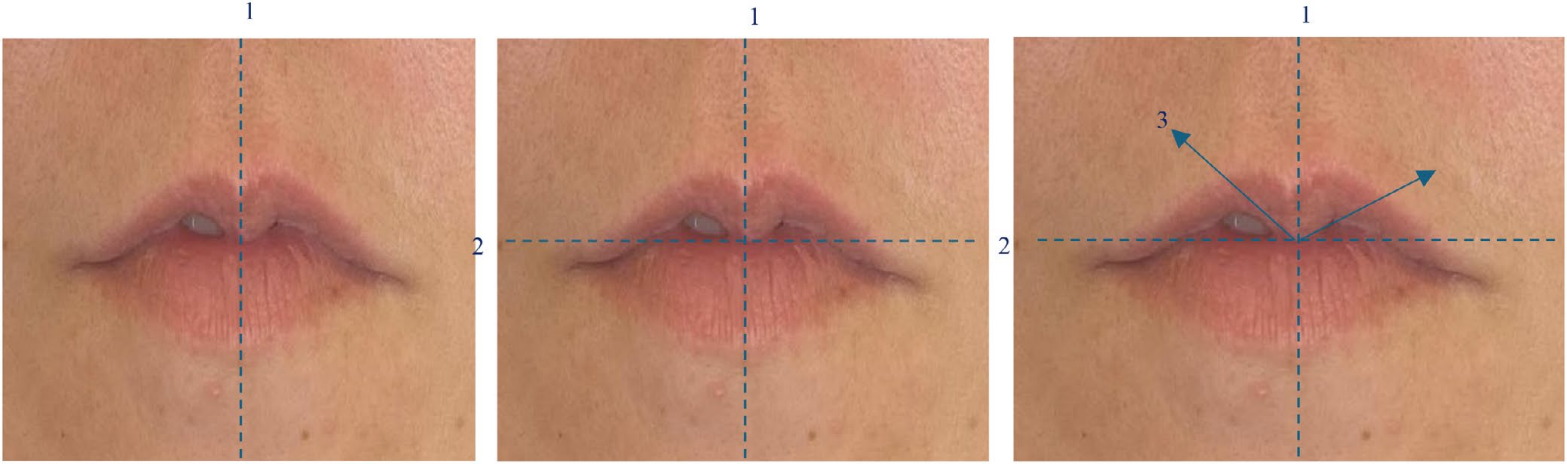
Sequential line drawing for characterization of M‐shaped lips.

M‐shaped lips were classified based on the angle formed between the vertical and horizontal midlines of the lips, observed, and measured using standardized frontal facial photographs. A specific description of the technique is shown in Figure 3. Briefly, *line 1*, a vertical line corresponding to the midline of the face, is drawn in line with the midpoint of the cupid's bow, crossing the area of the greatest projection of the central tubercle of the upper lip. Next, *line 2*, a perpendicular line corresponding to the area of the maximum projection of the central tubercle at the junction of the wet and dry mucosa, is drawn. Finally, *line 3* is drawn, originating from the intersection of *line 1* and *line 2*, extending to the point of least volume on the lateral tubercles. The angle between *lines 2* and *3* is measured using a goniometer or protractor. If the measurements differ, the greater angle to define the grades is used.

### 2.3 The Classification Was Categorized Into Three Severity Grades

Mild (grade I): Lips with an opening angle of up to 15°. These lips are characterized by the absence of teeth visible upon lip closure and are often amenable to treatment in a single session.

Moderate (grade II): Lips with an opening angle ranging from 16° to 25°, where teeth may become visible upon lip closure. The use of a cannula for the procedure is recommended for treatment, and two sessions are typically required for optimal results.

Severe (grade III): Lips with an opening angle greater than 25°, where teeth become visible upon lip closure. A cannula must be used to mitigate vascular compromise, and the procedure usually requires two or more sessions for comprehensive enhancement.

### 2.4 Treatment Protocol

Each patient underwent a pre‐treatment consultation where the specific classification was determined, and the treatment plan was designed accordingly. Digital photographs were taken to document each case pre‐ and post‐treatment.

### 2.5 Injection Techniques and Materials

Needles (30G) and cannulas (25G × 50 mm) were used for product placement and distribution. Aliaxin LV (IBSA Derma), an HA‐based filler approved for lip augmentation, was used. The product is characterized by its tan delta (flow) and plasticity, ensuring homogeneous diffusion in the lip mucosa and high resistance to mechanical stress. Aliaxin LV also boasts an optimal G' lift (stiffness) property, guaranteeing harmonious volumization [13, 14, 15]. Its rheological properties further contribute to proper lip volume without compromising a natural appearance or exposing the patient to stigmatization [13, 14, 15]. Topical anesthetic (EMLA cream) and infiltrative lidocaine 2% were used during the first procedure to manage pain and discomfort.

### 2.6 Session Details

**FIGURE 4:**
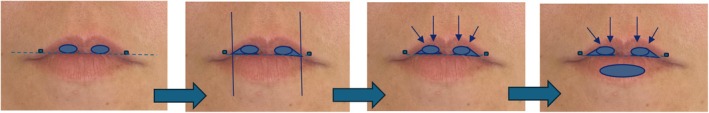
Steps for filler material deposition in M‐shaped lips.

First session: The primary goal was to establish the basic M‐shape and address the most significant deficiencies in lip symmetry and volume. The volume administered was tailored to the patient's anatomical needs but generally started with 0.7 mL. A 25G cannula was used, guided by *line 2* as previously described for classification. The cannula was inserted at the vermillion border on both sides. It was then traversed linearly 1 mm above the junction of the dry and wet mucosa, and the product, Aliaxin LV, was deposited in the area corresponding to the absent lateral tubercles to artificially create them using the HA filler. The product was deposited with a linear retrograde injection technique, taking care not to exceed the width of the nasal ala. To enhance the cupid's bow, a 31G needle was used to administer the product in a retrograde injection as follows: following the philtrum, the needle was inserted at the vermillion border up to 1 mm from the junction of the wet and dry mucosa, depositing the product with a retrograde technique. If deemed necessary to enhance the outcome, two additional retrograde deposits were placed 5 mm apart, converging at the initial application site along the first line. Volume was also added to the lower lip using the most appropriate technique (see Figure 4).

**FIGURE 5:**
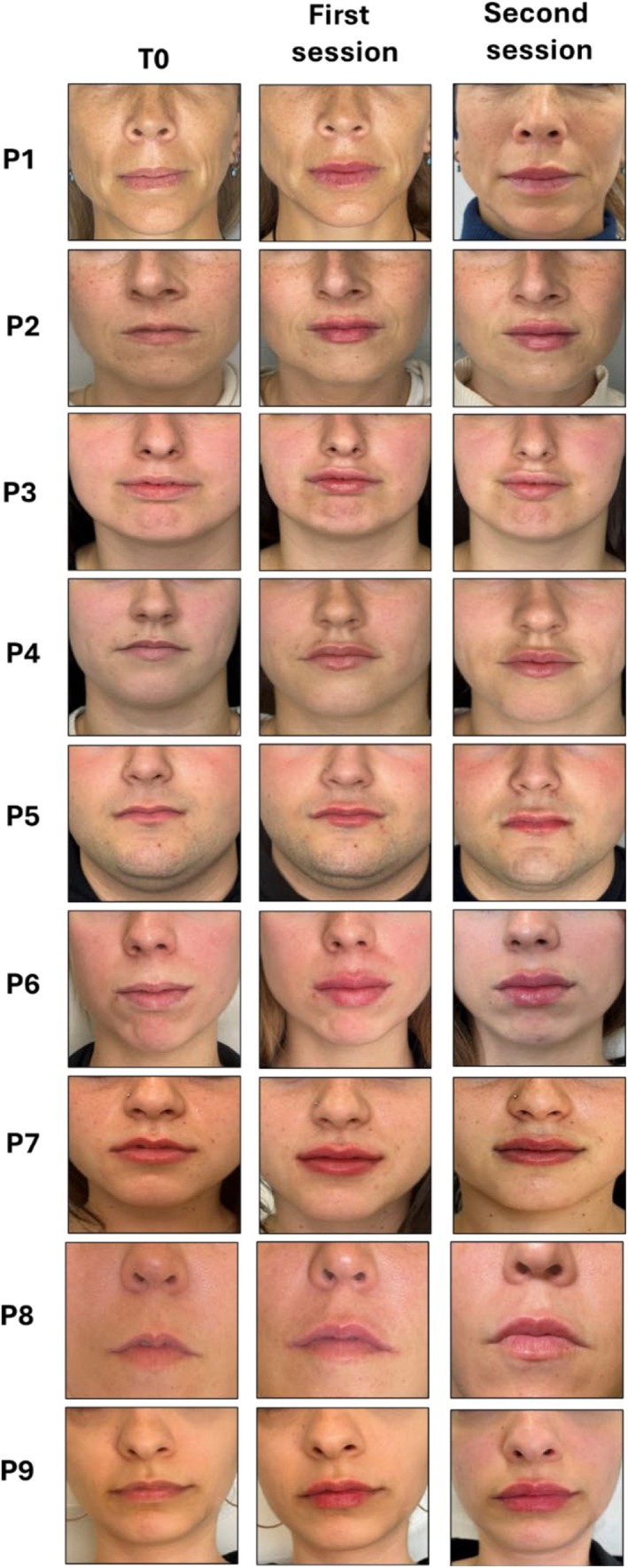
Photographs of study patients' lips before and after first session and second session of treatment.

Second session: This occurred 2 weeks after the initial treatment, focusing on refinement and adjustments. The additional volume (ranging from 0.2 mL to 0.4 mL) was administered based on the integration of the filler and the patient's response to the first session. Standardized photographs were taken at each session to assess the aesthetic outcomes and any adverse effects (Figure 5).

### 2.7 Documentation and Follow‐Up

Patients were followed up for a month after their last session to evaluate the immediate and short‐term outcomes of the treatment and their satisfaction.

### 2.8 Ethics

Following the principles of the Helsinki declaration, all patients received information about the product and procedure and signed an informed consent form for the procedure and for their anonymized clinical data to be used for scientific purposes.

## 3 Results

A total of nine patients with M‐shaped lips, aged between 20 and 45 years, were classified based on the angle formed between the vertical and horizontal midlines of the lips and treated with Aliaxin LV at different doses, depending on their specific requirements (Table 1). Most were females (89%) and only two had had previous cosmetic treatments. Despite the anatomical complexities associated with M‐shaped lips in lip augmentation procedures, we did not observe challenges such as filler displacement toward the white lip or vermilion border and migration toward the central tubercle of the upper lip after either the first treatment or the second. Additionally, there were no instances of visible Aliaxin LV clusters or inadequate integration with the surrounding tissue. The superficial placement of the artery due to reduced submucosal space, as described in M‐shaped lips, did not pose any complications in this study, and aesthetic outcomes were good (Figure 5). All patients were satisfied with the results of the intervention, reporting improved lip symmetry, volume, and overall aesthetic appearance.

**TABLE 1:** Summary of clinical data and treatment outcomes of the study participants.

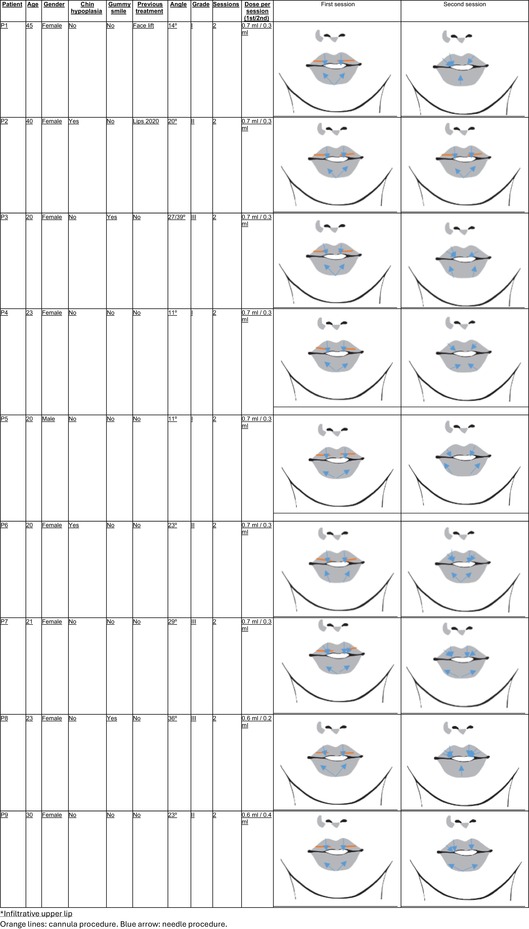

## 4 Discussion

In this case series, we propose simplifying the diagnosis of M‐shaped lips by introducing a new classification system. To date, there is a notable absence of literature or medical publications specifically addressing the identification, naming, or classification of this distinct anatomical variant. Our proposed classification, based on meticulous measurements of facial anatomical parameters, facilitates the precise placement of HA filler in optimal anatomical locations, thereby enhancing aesthetic outcomes and minimizing potential complications. This structured approach ensures tailored treatments for each patient, aiding clinicians in achieving consistently superior results.

Patients with M‐shaped lips often request aesthetic procedures to improve their appearance. The lips are a prominent facial feature and, as such, require meticulous attention to detail to avoid vascular complications and undesirable aesthetic outcomes, both of which are common concerns in lip augmentation procedures [1, 4, 5, 6, 16]. These possible complications are often associated with discomfort, which can directly affect patients' quality of life [3]. Although various anatomical guidelines have been proposed for preventing adverse outcomes [1, 9], the clinical complications that typically arise from either intravascular or extravascular injection of a filler product remain a significant risk.

Moreover, as aesthetic procedures are voluntary, it is crucial to fulfill patients' desires. A proper explanation of the treatment process, acknowledging the limitations of the outcome, and communicating realistic expectations and potential risks are mandatory to achieve good outcomes alongside the anatomical considerations. Using our proposed classification, HA filler is tailored to the anatomical situation of each patient, and the appropriate technique, cannula, and needle are selected to obtain better results and avoid long‐term deterioration.

Choosing the most suitable HA is a fundamental factor in correctly treating M‐shaped lips. Since we are increasing volume to upper lip tubercles that do not naturally exist, it is essential that the product has high tissue integration and high cohesiveness. Aliaxin LV is, therefore, the best filler option due to its rheological characteristics [13, 14, 15, 17]. It has an ideal tan delta (flow) of 0.21 and plasticity of 0.15, ensuring homogeneous diffusion within the labial mucosa and providing excellent resistance to mechanical stress [13], and its optimal G' or lift of 107 Pa guarantees harmonious volumization [13]. These rheological properties are essential for achieving correct lip volume without compromising a natural appearance or exposing the patient to stigmatization [13].

Our study has some limitations that must be acknowledged. First, considering that the sample size was relatively small, any generalization of results should be approached with caution. Secondly, the lack of diverse ethnic backgrounds among our participants could limit the applicability of the classification system across different populations. Moreover, the follow‐up period is too short to establish the effectiveness and safety of the system in long‐term use. In future studies, study populations should be larger and more diverse, and a longer follow‐up period is needed for better assessment of long‐term outcomes. Integrating advanced imaging techniques, such as ultrasound monitoring, would also offer more accurate and safer interventions.

On the other hand, this study offers a number of advantages, including our new classification of M‐shaped lips based on extremely detailed anatomical measurements. This method can be tailored to increase the safety of HA filler injections and avoid or reduce side effects, while increasing the aesthetic effect. Our study emphasizes patient safety and satisfaction, highlighting the importance of clear communication and fostering realistic expectations. It also forms a basis for future studies and eventual standardization of the methods of lip augmentation, making clinical practice more uniform and reliable.

This newly proposed M‐shaped lip classification system seems to hold great promise in terms of better aesthetic outcomes with fewer complications. Although more research is needed to establish its effectiveness and applicability, this new method will help advance the aesthetic treatment of lips and offer better care to patients undergoing these procedures.

## Conflicts of Interest

D.M.V. has received payment or honoraria for lectures, presentations, and speakers' bureaus from IBSA, Instituto Bioquimico Iberico SL. Aliaxin LV was provided by IBSA, Instituto Bioquimico Iberico SL.

## Data Availability

The data that support the findings of this study are available on request from the corresponding author. The data are not publicly available due to privacy or ethical restrictions.
